# Ethnic-racial identity affirmation: Validation in Aboriginal Australian children

**DOI:** 10.1371/journal.pone.0224736

**Published:** 2019-11-07

**Authors:** Davi Manzini Macedo, Pedro Ribeiro Santiago, Rachel M. Roberts, Lisa G. Smithers, Yin Paradies, Lisa M. Jamieson

**Affiliations:** 1 Indigenous Oral Health Unit, Adelaide Dental School, The University of Adelaide, Adelaide, Australia; 2 School of Psychology, The University of Adelaide, Adelaide, Australia; 3 BetterStart Child Health and Development Research Group, School of Public Health, The University of Adelaide, Adelaide, Australia; 4 Alfred Deakin Institute for Citizenship and Globalisation, Deakin University, Melbourne, Australia; University of Sao Paulo Medical School, BRAZIL

## Abstract

**Introduction:**

Positive attitudes towards ethnic-racial identity (ERI) is a key factor in Aboriginal Australian children’s development. The present study aims to offer evidence of construct and criterion validity, reliability, and measurement invariance of a brief measure of Aboriginal children’s ERI affirmation.

**Methods:**

Data was from 424 children aged 10–12 years (mean 10.5 years; SD 0.56) participating in the 8^th^ wave of the Longitudinal Study of Indigenous Children (LSIC). Information on ERI was obtained from 4 child-reported items. Sociodemographic characteristics and child social and emotional outcomes were caregiver-reported. A factorial structure was tested by Confirmatory Factor Analysis. The estimation method was weighted least squares with mean and variance adjusted test statistic (WLSMV). For reliability verification, the ordinal α and Ω hierarchical α were assessed. For construct validity, a generalized linear model with log-Poisson link estimated the association between ERI and children’s social and emotional outcomes. We hypothesized that children with positive ERI would have lower behavioural and emotional difficulties.

**Results:**

We found evidence of excellent fit for a unidimensional model of ERI affirmation after adjusting for correlated uniqueness between items 1 and 3 (χ^2^(2) = 0.06, p = 0.80; RMSEA = 0.000 [90% CI 0.000–0.080], p = 0.088; CFI = 1.000). Internal consistency reliability was considered adequate (ordinal α = 0.83; Ω hierarchical α = 0.72). The unidimensional model was shown to be invariant among boys and girls (Δχ^2^ (4) = 6.20, p = 0.18; ΔCFI = 0.000). Higher ERI was associated with lower risk of problematic scores (>17) on the SDQ (Risk Ratio_a_ = 0.91, 95% CI 0.64, 1.29).

**Discussion:**

The four LSIC items perform as a brief measure of Aboriginal children ERI affirmation among boys and girls. Results contribute much needed evidence for LSIC’s ongoing success and to future research on Aboriginal children’s development and wellbeing.

## Introduction

Ethnic and racial minorities’ feelings and beliefs about their ethnic and racial memberships have been a topic of interest in the social sciences for decades [1]. A recent effort to unify this field of research–the Ethnic and Racial Identity in the 21^st^ Century Study Group- has proposed the adoption of a meta-construct to refer to this process of self-understanding and self-categorization. The ethnic-racial identity (ERI) concept was proposed to represent the perception of belonging to a social group across ethnic and racial groups from different heritages, nationalities, cultural backgrounds, and socialization experiences [1].

The process of identification with one’s ethnic-racial group starts early in development. Children as young as five-years possess a sense of ERI and demonstrate that they explore, commit, and consolidate attitudes and preferences based on ethnic-racial membership [2–4]. During childhood ERI is associated with higher self-esteem, better adaptive behaviour and fewer externalizing and internalizing problems [3, 5]. Furthermore, positive attitudes and a sense of commitment to ERI are shown to exert a protective role against the effects of racism on racial minority children and adolescents’ wellbeing [6, 7]. The attitude component of ERI has been referred to as ERI affirmation and can be observed from an early age [8]. Attitudes towards ERI are central to wellbeing and mental health, as feelings towards social identities (e.g., gender, race-ethnicity, nationality) are decisive in perceptions of self-esteem and global self-worth [9, 10].

Aboriginal Australians are the descendants of the occupants of the Australian continent prior to European colonisation [11]. There are diverse communities of Aboriginal Australians with unique traditions, political systems, cultural characteristics, and languages, living all across the Australian territory (from metropolitan centres to remote communities) [11]. It is estimated that Aboriginal Australians comprise approximately 3.3% of the Australian population, corresponding to 798,365 inhabitants accordingly to the last Australian Bureau of Statistics census, dated from 2016 [12]. As a disadvantaged group in Australia, due to a history of colonization and dispossession [13], Aboriginal Australians face a range of social inequalities (e.g. lower educational attainment and income, poor access to health services) [11] and can experience discrimination from early ages [14, 15]. Discrepancies in their mental health and wellbeing are also documented. A recent report on Aboriginal youth wellbeing suggested one third of participants (33%) indicated experiencing high to very high levels of psychological distress, against 13% of their non-Indigenous counterparts. Alarmingly, suicide was identified as one of the leading causes of death among Aboriginal Australians aged 10–24 between 2011 and 2015. [16].

Nonetheless, approaches have been proposed emphasizing the resilience of Aboriginal people in facing the adversities that affects this population. The importance of culture, spirituality, connection to land, ancestry, kinship, and a sense of pride about being Aboriginal have been consistently reported as a central determinant of Aboriginal Australians’ health and social and emotional wellbeing across the lifespan [13, 17]. Research on Aboriginal perspectives of positive child development highlights the importance of a strong sense of attachment to culture and pride about their Aboriginal identity [14, 18]. Despite the relevance of ERI to both developmental psychology and the Aboriginal holistic perspective of health and development, limited data measuring this construct among Aboriginal children is currently available.

Footprints in Time: The Longitudinal Study of Indigenous Children (LSIC) is one of the few initiatives that have assessed ERI among Aboriginal children [19]. LSIC collects information on determinants of Aboriginal children’s development across a wide range of communities and environments, including more than 80 Aboriginal clans and tribal groups across Australia. [20]Nonetheless, there is no published evidence regarding the validity and reliability of the ERI items used in data collection. Therefore, the present study aims to evaluate the construct validity and reliability of the ERI items as a measure of content/attitudinal ERI. Our hypothesis is that the 4 items used provide a brief unidimensional psychological instrument of how Aboriginal children perceive and feel about their ethnic-racial membership. A unidimensional instrument is one which the responses to all items (or, alternatively, the covariance between items) can be explained by a single latent variable [21]. That is, all the items measure a single underlying construct. In practical terms, methods such as factor analysis can show whether an instrument is unidimensional by evaluating if a one-factor model is a good fit for the data (compared to other models such as two or three-factor models, for example) and checking if “all items have substantial factor loadings on a single factor” [22]. The psychometric analysis will evaluate: a) the factorial structure of the items; b) measurement invariance by gender; c) reliability; and d) criterion validity.

To the best of our knowledge, this is the first study to assess the efficacy of a brief instrument targeting an affective component of Aboriginal children ERI (ERI affirmation). Additionally, LSIC is a pioneer study due not only to its longevity, but the diversity of children and families represented and the integration of Aboriginal cultural values and perspectives in its design and data collection [19]. Therefore, the verification of the validity of the measures applied, especially when concerning an aspect of central importance for Aboriginal Australians, may aid in its continued success.

## Methods

### Study design

LSIC employs an accelerated cross-sequential design aimed to collect information on the first nine to ten years of Aboriginal children’s development in a six-year period. The study involves two cohorts. The B cohort includes children who were aged 0.5 to 2 years at wave 1. The K cohort consists of children aged 3.5 to 5 years at the beginning of the study. The content of the questionnaires is selected through consultation with working reference groups, community stakeholders from urban, regional and rural Indigenous communities, as well as academic institutions and government agencies [20]. Ethical approval for the content selection and data collection processes is obtained from the Human Research Ethics Committee of the Australian Institute of Aboriginal and Torres Strait Islander Studies [20]. LSIC waves occur annually between February and December. Data from waves 1 to 9 (2008–2016) is currently available upon application and a signed deed of license from the Australian Government Department of Social Services (DSS), the party responsible for conducting LSIC [23].

### Data collection procedures

A non-random purposive sample was recruited from records of Centrelink and Medicare Australia, welfare and health-assistance programs, respectively [24, 25]. Signed consent was obtained from the eligible families who agreed to participate. Participants were also recruited through informal means of communication such as local study promotion and personal communication among community members. Interviews were conducted by Department of Social Services Aboriginal and Torres Strait Islander Research Administration Officers [20]. In wave 1, over 1,680 interviews were conducted with children’s primary caregivers. A total of 1,255 interviews were conducted in wave 8 (2016), corresponding to an 87.2 retention rate from the previous wave [20]. Authors received permission to access de-identified data upon DSS’s authorization [23]

### Participants

Children in the K-Cohort participating in Wave 8 of LSIC were included in the analysis. Between both cohorts, there were 1,240 participating children. However, ERI was only assessed among the children in the K cohort (n = 496). Of those, 47 were excluded as caregivers did not authorize the research administration officers to administer the ERI affirmation items. Among the 449 children that responded to the measure, 9 were excluded due to missing values in at least one of the 4 items. Since our aim was to evaluate the validity and reliability of the 4-items for a specific age range, we focused on children aged 10–12 years (n = 435). Children aged 9 years (n = 5) or who had already turned 12 (n = 11) were removed due to small sample sizes. Our final sample thus comprised 424 Aboriginal children (51.3% males; mean age: 10.5 (SD 0.5) years).

### Measures

#### Ethnic-racial identity affirmation measure

A set of four child self-report items was used to assess participant’s ERI affirmation. All items had a 6-point Likert Scale response option, ranging from “Yes (Always)”, “Yes (Most of the time)”, “Sometimes (Fair bit)”, “Sometimes (Little bit)”, “No (Not much)”, “No (Never)”. Values from 1 to 6 were assigned to responses and reverse-coded so higher values would suggest higher ERI affirmation. Two other alternative response options were “Don’t know” and “Refused”, coded as missing. The 4-items were: 1) “I feel good about being Aboriginal and/or Torres Strait Islander in class”; 2) “I want to share (tell others) things about being Aboriginal and/or Torres Strait Islander in class”; 3) “I feel safe about being Aboriginal and/or Torres Strait Islander in class”; and 4) “I like people to know I am Aboriginal and/or Torres Strait Islander in class”.

The measure was selected by the LSIC team after consultation with the LSIC steering committee and community stakeholders, as a standard procedure adopted to guarantee community participation and the integration of Aboriginal cultural values and perspectives [20]. The original items are part of a measure to assess cultural and Aboriginal educational strategies [26]. The items were originally presented as the factor “Strength of Cultural Identity”. Two of the items were modified for use in LSIC. Item 3 was originally worded “I feel comfortable about my culture in class” and item 4 was “I am proud of my culture when I am in class”.

#### Socio-demographic characteristics

Information on participant’s age and sex was collected at wave 1 through an open and caregiver-reported question. For confounding adjustment in the criterion validity analysis, information on the family Level of Relative Isolation (LORI), and the index for Indigenous Socio-Economic Outcomes (IRISEO) were also used. The LORI is based on the Accessibility/Remoteness index of Australia and is a measure of remoteness that reflects distance to service centers. The LORI index is an area level indicator and it ranges from 1 to 5, from “no isolation”, which corresponds to metropolitan areas, to “extreme isolation” [27]. The IRISEO is calculated specifically for Aboriginal Australians and is an area-level measure of community socioeconomic disadvantage based on education, employment, income, and housing. It ranges from (1) disadvantaged to (10) advantaged [28].

#### Strengths and difficulties questionnaire (SDQ)

Child social and emotional outcomes were assessed by the caregiver’s version of the SDQ. The instrument is validated for use among 4 to 17 years old [29]. The SQD has been recently validated for Aboriginal children of this age range (4–17 years), displaying good psychometric properties and excellent overall reliability [30]. It assesses levels of emotional and behavioral difficulties in four domains: emotional difficulties, conduct problems, hyperactivity, and peer problems. Each domain is composed of five items with responses ranging from 0 “Not true” to 2 “Certainly true”. Examples of items are “often unhappy, depressed, or tearful” (emotional difficulties), “steals from home, school, or elsewhere” (conduct problems) and “restless, overactive, cannot stay sill for long” (hyperactivity). A score-range from 0 to 10 is obtained for each domain. A total score for emotional and behavioral difficulties is computed by summing the scores on the four domains (0–40). Higher scores indicate higher levels of difficulties that might represent risk for future clinical symptomatology [29].

### Statistical analysis

The first step of the analysis was a Confirmatory Factor Analysis (CFA) to evaluate the fit of the hypothesized one-factor model. The estimation method was weighted least squares (WLSMV) with mean and variance adjusted test statistic [31]. WLSMV estimation is recommended for use with non-normal distributions [32], such as the four *ordinal* ERI items, and skewed data [33]. Considering that the percentage of missing data in individual items was below 1%, multiple imputation would not be likely to change the results and listwise deletion was employed. Furthermore, WLSMV estimation with listwise deletion can be used when the amount of missing data is unsubstantial, producing unbiased estimates for the parameters and their standard errors [34].

The sample size used (n = 424) was considered adequate for our analytical purposes. In general, there are two guidelines for sample size requirements in CFA models: (1) the absolute sample size (N), in which N ^3^ 300 guarantees accurate parameters and fit statistics in WLSMV estimation [35]; and the relative sample size to number of estimated parameters (q), namely the N:q ratio, which should have a value above 10:1 [36]. In our study, considering that the most complex model had 25 estimated parameters (q = 25), the sample size requirements were achieved both in an absolute (n = 435) and relative (N:q = 17.4) sense. Model fit was evaluated with the scaled χ^2^, in addition to the scaled Comparative Fit Index (CFI) and the scaled Root Mean Square Error of Approximation (RMSEA). Values of CFI ≥ 0.96 and RMSEA ≤ 0.5 indicated good fit [37]. Values of RMSEA ˃ 1.0 were considered to be indicative of poor fit [38] and the hypothesis of close-fit (RMSEA ≤ 0.5) was evaluated [39].

In case of a poor fitting model, model re-specifications were conducted by the evaluation of standardized residual correlations, modification indices (MI) and standardized expected parameter change (SEPC) [40]. After a model was established, we proceeded to evaluate measurement invariance by gender to check whether the items functioned differently between boys and girls. Testing invariance by gender intends to account for possible differences among boys and girls regarding transmission of cultural practices and racial socialization, which can influence children’s attitudes towards ERI [41, 42]. Configural, metric and scalar invariance were evaluated with χ2 [43]. In the event χ2 was statistically significant, the ΔCFI [44] was used, with invariance being assumed when the CFI values do not vary above 0.002 points between models. Finally, reliability was evaluated with the ordinal **α** [45]. The use of the ordinal **α** is required since Cronbach’s **α** [46] underestimates reliability in ordinal items, such as Likert scales [45]. Reliability above 0.80 is usually deemed acceptable for validation studies such as ours [47]. Analyses were conducted in R software [48], R packages lavaan 0.6–2 [49] and semTools [50].

For the criterion validity analysis, the association between ERI affirmation and child total emotional and behavioural difficulties was tested. Our hypothesis was that children with high ERI affirmation would be at decreased risk for the onset of emotional and behavioural difficulties, as per the associations of ERI and positive developmental outcomes among ethnic-racial minority children [51]. Generalized linear models were preferred as the specification of the link function allows accommodation of non-normal distributions and skewed data [52]. We estimated risk ratios as a measure of the effect by testing a generalized linear model with a log-Poisson link and robust errors (model 2). The log Poisson link was chosen as we aimed for risk ratios as effect-measures and robust errors were specified to generate unbiased effect estimates in case of model misspecification [53]. The exposure and outcome variables were dichotomized. ERI affirmation was divided into “high” and “low”. The high ERI affirmation category was composed of the children who endorsed “Yes (Always)” and “Yes (Most of the time)” to all four items of ERI affirmation (≥20). The SDQ total score was dichotomized in “high difficulties” (scores ≥17), and “low difficulties” (scores ≤ 17) [54]. The two models were adjusted for child age and sex, and family LORI, and IRISEO, confounding selected as per associations reported in the literature among these sociodemographics and both ERI and wellbeing [42, 55, 56]. The models were tested with 419 children, as five children had no information on the SDQ total score, LORI and IRISEO variables.

## Results

The first model tested was the one-factor model and the fit indices provided mixed evidence regarding model fit (Table 1). The sample of the fitted model was 424 participants. Although the CFI was above the threshold of 0.96, a statistically significant χ2 and a RMSEA of 0.128 were observed. Additionally, the p-value of 0.01 indicates that the hypothesis of close-fit (RMSEA<0.5) was rejected. We explored possible adjustments to improve the model by carrying specification searches. The examination of the standardized expected parameter changes (SEPC) showed that items 1 and 3 residuals had a correlation of 0.65. Therefore, we observed that correlated uniqueness between two items could be limiting the fit of the data to the confirmatory structure tested.

**Table 1:** Fit indices for the two unidimensional models of ERI affirmation.

Model	χ^2^	*df*	*p-*value	RMSEA	90% CI	p-close	CFI
Model 1	15.89	2	0.000	0.128	0.075–0.190	0.01	0.983
Model 2	0.121	1	0.72	0.000	0.000–0.091	0.837	1.000

We proceeded to test a second model that accounted for correlated uniqueness between items 1 and 3. The model had an excellent fit to the data (χ^2^ (1) = 0.121, p = 0.72). The RMSEA value of 0.000–90% CI [0.000, 0.091] for this second model suggested that the covariance of item responses was sufficiently explained by the underlying one-factor model specified. The 90% CI shows that the range of compatible values with the model are mostly below the threshold of 0.05, although values above it could also be compatible. Nonetheless, testing for the alternative hypothesis that the RMSEA value falls below the value of 0.05 resulted in a p-value of 0.83, suggesting that the hypothesis of close-fit should not be rejected. The CFI value was above the 0.96 threshold, suggesting excellent model fit. The interpretation of the fit indices and the available CI and significance tests suggest there is evidence of construct validity for this one-factor model of ERI affirmation. Comparisons between the fit indices from the two CFA models tested are showed in Table 1. Table 2 presents the second model items’ loadings on the underlying ERI affirmation factor.

**Table 2:** Item loading estimates of the two unidimensional models of ERI affirmation.

Items	Model 1		Model 2	
	Estimate	95% CI	Estimate	95% CI
1.“I feel good about being Aboriginal and/or Torres Strait Islander in class”	0.77	(0.70, 0.85)	0.66	(0.56, 0.76)
2.“I want to share (tell others) things about being Aboriginal and/or Torres Strait Islander in class”	0.63	(0.56, 0.73)	0.65	(0.57, 0.73)
3.“I feel safe about being Aboriginal and/or Torres Strait Islander in class”	0.82	(0.74. 0.88)	0.72	(0.64, 0.81)
4.“I like people to know I am Aboriginal and/or Torres Strait Islander in class”	0.79	(0.73, 0.87)	0.86	(0.79, 0.94)

**Fig 1:**
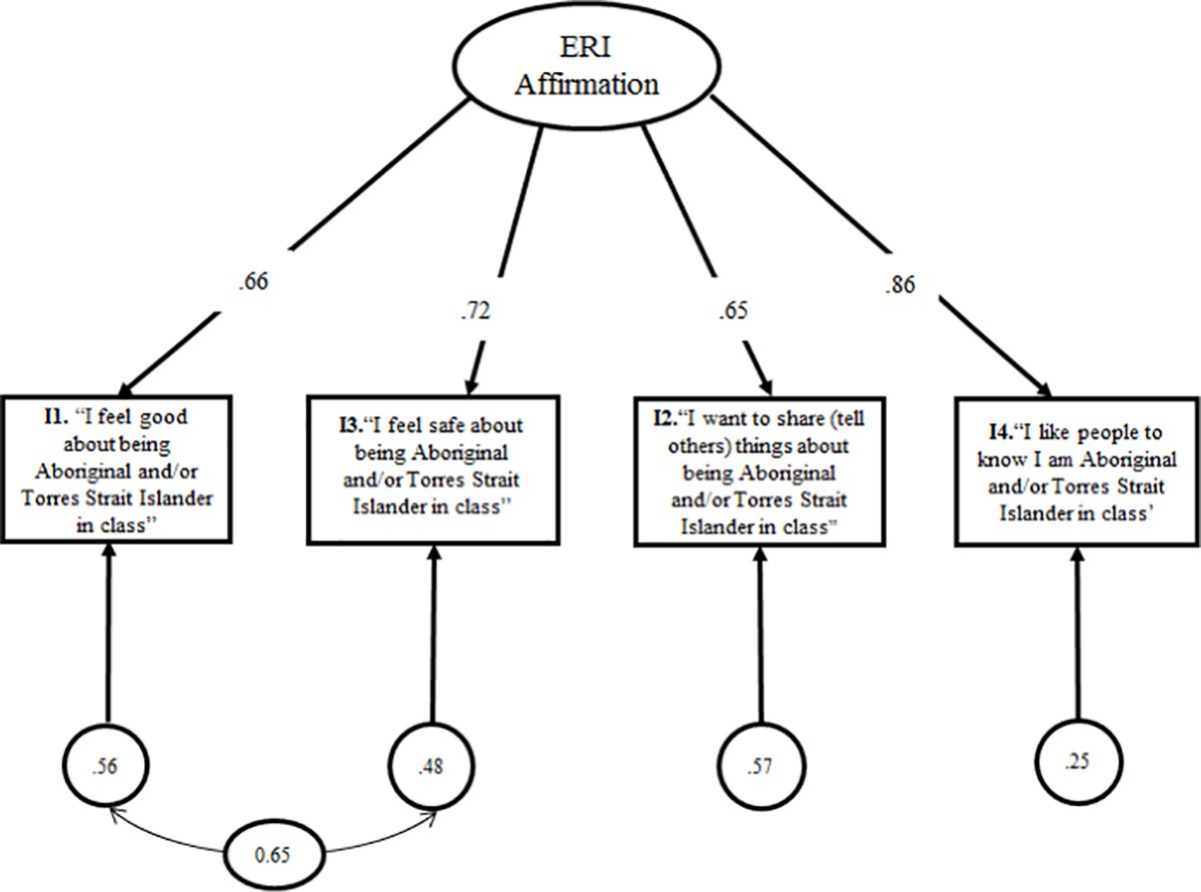
Unidmensional structural model of ethnic-racial identity affirmation accouting for correlated uniqueness.

The unidmensional structural model of ethnic-racial identity affirmation is illustrated in Fig 1, accounting for correlated uniqueness between items 1 and 3. Internal consistency reliability for the measure was considered adequate, as was the ordinal α of 0.83. The Ω hierarchical α, a reliability index that accounts for correlated uniqueness among items, was also assessed and its value of 0.72 was considered satisfactory [57]. Finally, the analysis of measurement invariance indicated scalar invariance (Δχ^2^ (4) = 8.86, p = 0.78; ΔCFI = 0.000), demonstrating that the unidimensional model is invariant among boys and girls (Table 3).

**Table 3:** Fit statistics for measurement invariance according to gender.

Model	χ^2^	*df*	*p-*value	RMSEA	90% CI	CFI	Δ χ2 (df)	*p-*value	Δ CFI
*Configural*	0.25	2	0.88	0.000	[0.000, 0.066]	1.000	-	-	-
*Metric*	2.16	5	0.83	0.000	[0.000, 0.057]	1.000	2.10 (3)	0.55	0.000
*Scalar*	8.86	13	0.78	0.000	[0.000, 0.046]	1.000	8.26 (8)	0.41	0.000

Note. χ2 = chi-square; df = degrees of freedom; RMSEA = root mean square error of approximation; CI = confidence interval; CFI = comparative fit index; Δ χ2 (df) = chi-square difference and degrees of freedom; Δ CFI = CFI difference. The χ2 column reports scaled χ2. Δ χ2 (df) is a function of standard (not the scaled) χ2 statistics.

The next step of the analysis was the criterion validity. The results of the generalized linear model (n = 419) confirmed the hypothesis tested. Results showed that children with high ERI affirmation had a 9% decreased risk for presenting high SDQ scores (RR_a_ = 0.91, 95% CI 0.64, 1.29). The CI, however, showed that values above 1 could be compatible with the model, affecting the precision of the effect-estimate. Nonetheless, the results indicated that ERI affirmation had a protective effect for the onset of emotional and behavioural difficulties over and above levels of age, sex, geographical location, and socio-economic status. This suggests evidence of criterion validity of the ERI affirmation measure tested. Table 4 includes the frequency distribution of the exposure, outcomes, and confounding variables included in the analysis.

**Table 4:** Participant’s characteristics (n = 419).

Characteristic	Prevalence (95%CI)	n =
**Child Age (years)**		
10	47.0 (42.7, 52.3)	199
11	52.5 (47.7, 57.2)	220
Mean(SD)	10.5 (0.5)	
**Gender**		
Male	51.3 (46.5, 56.0)	215
Female	48.7 (44.0, 53.5)	204
**ERI affirmation**		
High ERI affirmation (≥20)	49.4 (44.6, 54.2)	207
Low ERI affirmation (<20)	50.5 (45.8, 55.4)	212
Mean (SD)	20.6 (3.6)	
**Emotional and Behavioural difficulties**		
Low difficulties (<17)	76.8 (72.5, 80.6)	322
High difficulties (≥17)	23.1 (19.3, 27.4)	97
Mean (SD)	10.4 (5.9)	
**Level of Relative Isolation (LORI)**		
None	28.9 (24.7, 32.4)	121
Low	55.1 (50.3, 59.8)	231
Moderate	7.6 (5.4, 10.6)	32
High/Extreme	8.3 (6.0, 11.4)	35
**Indigenous Index of Socioeconomic Outcomes (IRISEO)**		
Mean (SD)	5.8 (2.1)	

## Discussion

The CFA analysis provided evidence of construct validity that the brief measure of ERI affirmation works as a unidimensional scale among Australian Aboriginal children aged 10–12 years. The ordinal **α** and Ω hierarchical provided evidence that internal consistency reliability was adequate [47, 57]. In addition, the results of the generalized linear model tested contributes to evidence of criterion validity. The association between ERI affirmation and children’s emotional and behavioural difficulties reflect literature on the protective effect of positive attitudes towards ERI on the wellbeing of ethnic-racial minorities, including Indigenous youth from the U.S., Canada, and New Zealand [5, 58–60]. It also reflects Aboriginal Australians’ perspective on the importance of pride (positive attitudes) over ERI for positive Aboriginal children’s health and development [14, 18]. Such results contributes to the necessary evidence for research based on ERI data from LSIC, as it demonstrates that ERI affirmation is being assessed with a valid and reliable measure.

The initial model tested was a unidimensional model and the evidence regarding model fit was mixed. The results indicated strong correlated uniqueness between items 1 and 3. MacCallum, Roznowski [61] have discussed additional parameters due to specification searches, such as correlated uniqueness, and recommend that these parameters should be included only when justified by the theoretical background of the construct [62]. In our study, such a theoretical justification exists. The strong correlation between items 1 and 3 reflects the association between positive in-group attitudes (“I feel good about being Aboriginal and/or Torres Strait Islander in class”) and the levels of cultural safety perceived by respondents (“I feel safe about being Aboriginal or Torres-Strait Islander in class”). The perception of one’s social environment as accepting of cultural diversity might be linked, for example, to reduced experiences of racial discrimination and more positive experiences of ERI expression. Promoting cultural safety features as a key factor in improving Aboriginal health, education, and community wellness [63, 64]. For example, perceptions of cultural respect, peer acceptance of ERI, community involvement, and teacher’s cultural sensitivity–all contribute to a culturally safe environment—and are associated with less school absenteeism, higher classroom participation, and importance placed on school among Aboriginal students [26, 65].

After the inclusion of the correlated uniqueness between items 1 and 3, the model was correctly specified and achieved excellent fit. Therefore, the final measurement model was a unidimensional model *with* correlated uniqueness between items 1 and 3. The unidimensionality of the ERI items (i.e. the four items constitute a one-factor model) after the inclusion of the correlated uniqueness indicates that, although feelings of safety and positive experiences regarding ERI are more highly correlated with each other than others aspects of the construct (e.g., “I want to share (tell others) things about being Aboriginal and/or Torres Strait Islander in class”), the four items measure a single construct. That is, although the four items measure four distinct attitudes towards ERI ((1) feeling good about being Aboriginal and Torres Strait Islander; (2) wanting to share things about being Aboriginal and Torres Strait Islander; (3) feeling safe about being Aboriginal and Torres Strait Islander; and (4) liking people to know that they are Aboriginal and Torres Strait Islander), the CFA indicated that these attitudes constitute the broader construct of ERI affirmation. These findings are consistent with previous psychometric studies of ERI measures showing ERI affirmation as a distinct construct that encompasses several attitudes towards ERI (e.g. “I feel negatively about my ethnicity”) [8]. Finally, one practical implication of the four items measuring a common underlying construct is that item scores can be summated to create a total score [22] and this total score provides a measure of ERI affirmation.

To the best of our knowledge, there is just one validated scale to assess Aboriginal Australian children’s ERI [66]. The scale, however, focuses on exploration of cultural practices (knowledge of Aboriginal culture) and salience of racial identity, with no specific assessment of attitudes towards ERI [66]. The 40-item-length of the scale might also limit its applicability to large-scale studies such as LSIC. Here we provide evidence for a measure that’s strength resides in its brevity and specificity of content. This permits its inclusion in surveys desiring a holistic and comprehensive perspective of Aboriginal children’s wellbeing. The specificity of the measure reflects the debate on the importance of clearly defining which ERI component is being assessed [1]. Such accuracy might permit researchers to investigate how affective components of ERI relate, for example, to exploration of cultural practices and levels of commitment to one’s ERI (ERI processes) later in development [1, 8, 67, 68]. These distinctions might shed light onto how ERI develops among Aboriginal children and how the interplay between ERI processes and content relates to racial socialization processes and discrimination [67, 69].

Finally, investing on promotion of positive development from early age might assist in reducing health inequalities among Aboriginal children and youth and their non-Indigenous counterparts [16]. Due to its centrality to positive development and wellbeing, promotion of positive ERI attitudes might protect Aboriginal children against adversity and increase wellbeing [58, 70]. Research on evaluation of programs whose purpose is to increase the social and emotional wellbeing of Aboriginal children is still limited [71]. However, there is evidence of the efficacy of school-based interventions designed to increase affirmative ERI among other ethnic-minority youth (e.g., Latin, African, and Native-Americans), with reported effects on wellbeing and learning outcomes [72, 73]. Valid and reliable measurement of ERI can assist at baseline measurement and monitoring of outcomes for future interventions targeting cultural socialization and promotion of ERI in the Aboriginal Australian context [73].

We conclude that the LSIC items tested work as a brief measure of ERI affirmation by providing evidence of construct and criterion validity on a sample of 424 Aboriginal children aged 10 to 12 years. We recognise the limitation of not using a representative sample of the Aboriginal Australian children population. Nonetheless, the sample size used was considered sufficient for the analytical purposes of this study [35]. It is also noticeable that the LSIC is possibly the largest currently available source of information on ERI and other determinants of this population health and wellbeing [20]. The LSIC team has been committed to involving community stakeholders and field specialists in the selection of content and data collection procedures. The ERI items used were based on previous work on Aboriginal perspectives of wellbeing, which further contributes to the content validity of the measure. This is one of the few empirical demonstrations of the psychometric properties of a measure assessing components of Aboriginal children ERI. As such, it contributes to the development of this area of research in the Aboriginal Australian context.
